# Non-farm employment, natural resource extraction, and poverty: evidence from household data for rural Vietnam

**DOI:** 10.1007/s10668-022-02391-7

**Published:** 2022-05-26

**Authors:** Manh Hung Do, Trung Thanh Nguyen, George Halkos, Ulrike Grote

**Affiliations:** 1 Institute for Environmental Economics and World Trade, Leibniz University Hannover, Königsworther Platz 1, 30167 Hannover, Germany; 2 Department of Economics, University of Thessaly, 38333 Volos, Greece

**Keywords:** Rural livelihood, Heckman selection model, Simultaneous regression model, Instrumental variable, Q57, Q12, R20

## Abstract

Natural resources are important in sustaining the livelihoods of rural households and the environment. However, over-exploitation is causing an alarming depletion of natural resources in many developing countries. At the same time, rapid economic growth has created non-farm employment opportunities for local people. In this context, examining the interrelationship between non-farm employment and natural resource extraction provides useful information for reducing resource extraction and improving rural households’ welfare. In this study, we use a dataset of 1780 identical households from three survey waves undertaken in 2010, 2013, and 2016 in Vietnam to (i) identify the determinants of rural households’ participation in non-farm activities, (ii) examine the interrelationship between non-farm employment and natural resource extraction, and (iii) investigate the impact of non-farm employment on rural households’ welfare. The findings from pooled sample estimations reveal that (i) cable internet at home and rural road quality positively affect households’ decisions to participate in non-farm employment; (ii) non-farm income and income from natural resource extraction have a negative association; and (iii) non-farm income significantly contributes to poverty reduction in both relative and absolute terms. Our findings suggest that improved provision of non-farm opportunities and increased investment in infrastructure and telecommunication are needed to improve rural households’ welfare and consequently reduce their natural resource exploitation.

## Introduction

Natural resources, i.e. from rivers and forests, are scarce but play an essentially important role for humans and the environment. The extraction of natural resources is one of the principal livelihood strategies of rural households in developing countries, providing various products such as food, fuels, timber and non-timber forest products for home consumption and additional income from sales for rural households (Angelsen et al., 2014; Nguyen et al., 2018a). Further, the income from extraction of natural resources acts as a safety net in response to unexpected events such as illness and climate calamities (Nguyen et al., 2020; Wunder et al., 2014).

Empirical evidence from previous studies shows that the poor depend more on the extraction of natural resources as other livelihood alternatives available to them are much more limited than those to the rich (Kabubo‐Mariara, 2013). Therefore, maintaining a certain stock of natural resources is especially important for the poor. However, over-exploitation is causing a rapid depletion of natural resources in many developing countries and posing a major threat to the local environment and ecosystem. Although not all types of natural extraction would lead to over-exploitation, some extraction practices are causing serious problems to the ecosystems (Brodie et al., 2015). According to the Food and Agriculture Organisation (FAO) (2020a), global forests faced a loss of roughly 178 million hectares (ha) between 1990 and 2020. In 2019, the annual wood removals around the globe reached 3.97 billion m^3^ and 49% of this amount was for wood fuel (FAO, 2020b). Furthermore, degraded ecosystems caused by over-exploitation might not be able to fully recover and could have long-lasting effects on the future access to natural resources and ecosystem services (Lampert, 2019). Hence, fighting against the over-exploitation of natural resources and, at the same time, providing poor households with alternative livelihood opportunities are important for enhancing sustainable development and reducing poverty.

Finding livelihood alternatives to address the problem of the unsustainable extraction of natural resources is not an easy task. Some previous evidence suggests agricultural intensification to increase farm productivity and farm income, consequently reducing pressure on natural resources. However, agricultural intensification associated with an increase in chemical fertiliser and pesticide use has been found to damage the environment and human health (Nguyen et al., 2012, 2022). Other research suggests migration as an alternative livelihood strategy (Nguyen, Raabe, et al., 2015). In some rapidly growing economies, a higher demand for labour in urban centres and industrial zones attracts rural labourers to migrate and then send remittances to their rural origins; but some evidence shows that remittances might contribute to the enhancement of natural resource extraction (Bierkamp et al., 2021; López-Feldman & Chávez, 2017). In addition, an adverse shock as the Covid-19 pandemic has shown that the life of migrants in cities is also vulnerable. Millions of migrant workers were forced to return to their rural villages due to the lock-downs in urban centres leading to their job loss (Waibel et al., 2020). In this regard, non-farm employment such as wage-employment and self-employment in rural villages can play an important role in reducing natural resource extraction and improving household’s welfare. However, little attention has been paid to the interrelationship between rural non-farm employment and natural resource extraction in the literature.

Against this background, we aim to address the following questions: (i) What are the factors driving rural households to participate in rural non-farm employment? (ii) How is the interrelationship between rural non-farm employment and extraction of natural resources by rural households? And (iii) what are the impacts of rural non-farm employment on welfare, especially on poverty? Answers to these questions provide important insights on how to improve the livelihoods of rural households and to enhance natural resource conservation. They can also support us to achieve a number of the global Sustainable Development Goals (SDGs), namely zero poverty (SDG 1), sustainable cities and communities (SDG 11), and climate action (SDG 13) (Halkos & Gkampoura, 2021).

We focus our analysis on rural Vietnam. This country is well known for its rapid economic growth but has a high share of rural population. It has also been struggling to preserve its natural resource base and to reduce rural poverty. We use a dataset of rural households and villages from three survey waves undertaken in 2010, 2013, and 2016. We employ a two-step Heckman selection model to the first question, a simultaneous equation model to the second question, and an instrumental variable (IV) model to the third one. We undertake different statistical tests to validate our econometric specifications. Our findings reveal that cable internet at home and road quality positively affect households’ decisions to participate in non-farm employment and non-farm income; non-farm income and natural extraction income have a negative association; and non-farm income is significantly contributing to reducing both relative and absolute poverty in rural Vietnam.

The remaining of this paper is organised as follows. Section 2 describes the conceptual framework of our study. Section 3 introduces the data. Section 4 explains our estimation strategy. Section 5 presents the estimation results and discusses the findings. Section 6 summarises and concludes with policy implications.

## Conceptual framework

**Fig. 1:**
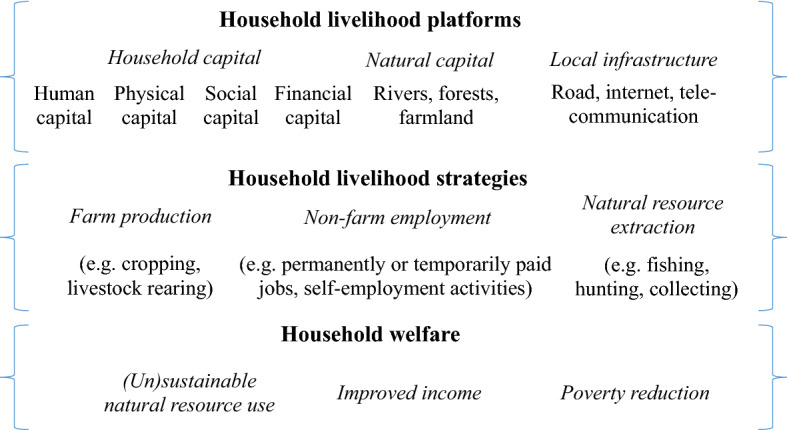
Conceptual framework of household livelihood analysis ( modified from Ashley & Carney, 1999; Nguyen, Do, et al., 2015; Soltani et al., 2012)

A household in a rural area of a developing country has various livelihood strategies to make its living. To explain the household’s choice of livelihood strategies, we employ the sustainable livelihood framework proposed by Ashley and Carney (1999) as the conceptual framework for our study (Fig. 1). The framework consists of three key components: (i) platforms for household livelihoods, (ii) household’s choice of livelihood strategies, and (iii) livelihood outcomes. The first component includes human capital (e.g. labourers and education), physical capital (e.g. productive equipment), social capital (e.g. social network), and financial capital (e.g. assets) and natural capital (e.g. rivers or forests for extraction) as well as local infrastructure (e.g. good roads and telecommunication). The second component illustrates the livelihood strategies or activities undertaken by the household based on the first component. It includes farm production (e.g. crop and livestock production), non-farm employment, or natural resource extraction (Do et al., 2019; Nguyen, Do, et al., 2015). The last component displays the outcomes of the second component in terms of the household’s welfare (i.e. income and poverty status) and the stock of natural resources. For the welfare of rural households, we focus on the poverty status due to its prevalence in rural areas and the dependence of the poor on natural resources.

To specify further the household’s choice of livelihood strategies, we use the model of labour allocation for household income maximisation (Weersink et al., 1998). We assume that each household has a fixed amount of labour (denoted as L), and they might allocate their labour to farming activities (*l*_*farm*_), extraction activities (*l*_*extract*_), non-farm activities (*l*_*non-farm*_), and other income-related activities. The return of labour is denoted as π. Therefore, the maximisation of household income ($\Pi )$ can be specified as follows:

1 $$\underset{{l}_{\mathrm{farm}},{l}_{\mathrm{extract}}, {\mathrm{l}}_{\mathrm{non}-\mathrm{farm}}}{\mathrm{max}}\Pi ={\pi }_{\mathrm{farm}}{l}_{\mathrm{farm}}+{\pi }_{\mathrm{extract}}{l}_{\mathrm{extract}}+{\pi }_{\mathrm{non}-\mathrm{farm}}{l}_{\mathrm{non}-\mathrm{farm}}+\mathrm{\rm P}$$

Subject to:

2 $${l}_{\mathrm{farm}}+{l}_{\mathrm{extract}}+{l}_{\mathrm{non}-\mathrm{farm}}\le L$$

3 $${l}_{\mathrm{farm},}{l}_{\mathrm{extract}},{l}_{\mathrm{non}-\mathrm{farm}}\ge 0$$

In Eq. (1), the income of the household includes four sources: (i) farm production (the return per labourer from farm production as ${\pi }_{\mathrm{farm}}$); (ii) natural resource extraction (the return per labourer from extraction activities as ${\pi }_{\mathrm{extract}}$); (iii) non-farm employment including wage-employment or self-employment activities (the return per labourer from non-farm activities as ${\pi }_{\mathrm{non}-\mathrm{farm}}$); and (iv) transfer incomes such as remittances or public transfers from the government (P). As for rural households, especially the poor, farming is essential to meet the subsistence needs for survival, it is chosen by default. The remaining issue is the choice between non-farm employment and natural resource extraction.

The interrelationship between natural resource extraction and non-farm activities is thus reflected by the constraint ${l}_{\mathrm{non}-\mathrm{farm}}=L-{l}_{\mathrm{extract}}-{l}_{\mathrm{farm}}$ Eq. (2), which leads to the simultaneity between non-farm income and natural resource extraction income. Hence, the estimation of these two income functions (for non-farm income and for natural resource extraction income) must simultaneously be undertaken.

The empirical evidence on the interrelationship between natural resource extraction and non-farm activities and the impacts of non-farm income on poverty in developing countries is rather scarce. Therefore, our study aims to fill the following research gaps. First, we examine the interrelationship between incomes from non-farm employment and resource extraction activities. We are the first to use an appropriate method to account for the simultaneity of non-farm income and resource extraction income. Second, we contribute to the investigation of livelihood alternatives to address the problem of the unsustainable extraction of natural resources. The results from our study provide solid evidence on helping rural households to reduce the over-exploitation of natural resources by stimulating alternative livelihoods in developing countries. Last, there are only a few studies on the effects of non-farm income on food security, food poverty, and vulnerability (e.g. Bui & Hoang, 2021; Do et al., 2019). The results from our study are expected to shed further light on the correlation of non-farm employment and poverty in developing countries.

## Data and descriptive summary

**Fig. 2:**
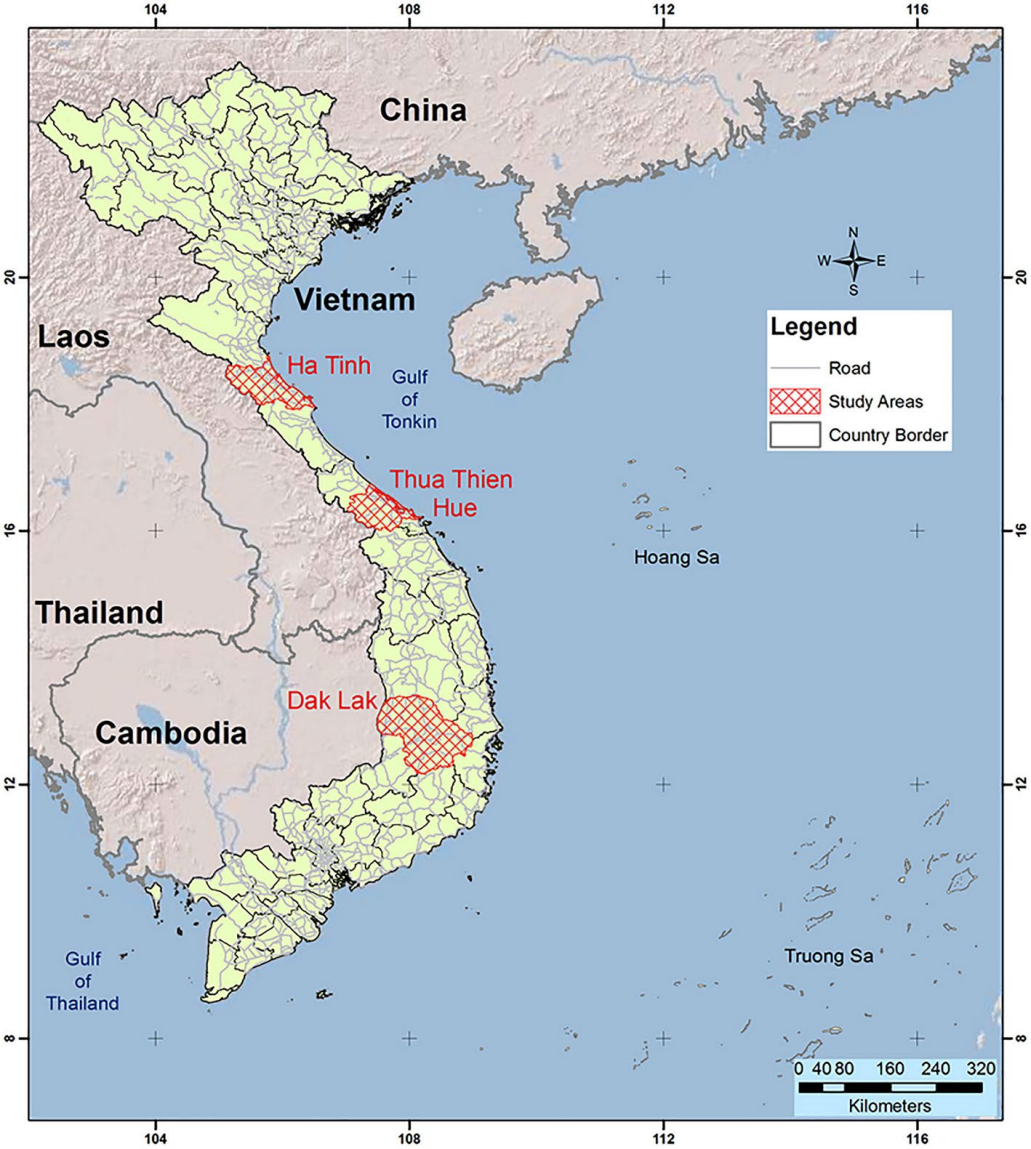
Study sites of the TVSEP project in Vietnam ( *Source*: Nguyen et al., 2021)

We use the data for Vietnam from the “*Thailand Vietnam Socio-Economic Panel (TVSEP): Poverty dynamics and sustainable development: A long-term panel project in Thailand and Vietnam*” funded by the German Research Foundation (DFG FOR 756/2) (see www.tvsep.de for the project’s website). The TVSEP data include about 2,200 households from 220 villages in three provinces in Vietnam, namely Ha Tinh, Thua Thien Hue, and Dak Lak (Fig. 2). The sampling method of rural household surveys under the TVSEP project followed a three-stage random sampling approach from commune, village, and household levels. At the first stage (selection of communes), the population share of communes in the districts was applied for weighting the selection of communes—the administrative system of Vietnam starts from provinces/municipalities (the highest level) to districts, communes, and villages (the lowest level). At the second stage (selection of villages), villages were sampled with a probability proportional to the size of the population in the communes. At the third stage, 10 households in each village were randomly selected from a list of all households in the sampled villages with equal probabilities (see Hardeweg et al. (2013) for detailed explanations of survey design and data collection).

In each wave, enumerators were carefully selected with an emphasis on their practical experience in carrying out rural household surveys. They then received intensive training before the surveys. During the surveys, each enumerator conducted face-to-face interviews at the respondent’s house. Each interview had an average duration of two and a half hours. The collected data went through three careful checking and cross-checking stages for any inconsistent and implausible information by (1) team leaders (at the spot), (2) data checking assistants, and (3) headquarter staff. If there was missing or implausible information, the responsible enumerator had to correct it by calling or re-visiting the household.

The TVSEP data have been collected from two structured questionnaires, one for the household heads and the other one for the village heads. The household questionnaire collects information on individual household members (e.g. age, education, health, and employment status), their livelihood strategies and household income (farm production including crop and livestock production, non-farm employment, and natural resource extraction), household expenditure, and remittances. The income data was collected for the past 12-months reference period. Along with the current values, the monetary values in the TVSEP dataset are converted to international dollars using Purchasing Power Parity (PPP$) and adjusted to 2005 prices for further comparison. The household head is usually the respondent of the interview. The village questionnaire captures village-related characteristics such as road conditions, number of enterprises in the villages, distances from the villages to district centre and province centre. In this study, we employ a dataset of 1780 households from 218 villages in three years (2010, 2013, and 2016) as they provide equal time gaps between the surveys (every three years). Compared with the number of successful interviews in the original sample in 2007, our reduced sample has an attrition rate of less than 5% per wave.

Hence, the final sample includes 5340 observations (1780 households from three surveys). (Detailed definition and measurement of household and village characteristics are presented in Appendix Table 6.) To provide a detailed overview of the household and village characteristics, the descriptive summaries of the data are presented on three different aspects: the households’ participation in non-farm activities (Table 1), their participation in extraction activities (Appendix Table 7), and by year (Appendix Table 8).

**Table 1:** Descriptive summary of household characteristics by participation in non-farm activities

	2010		Statistical test	2013		Statistical test	2016		Statistical test
	No (*n* = 525)	Yes (*n* = 1255)		No (*n* = 474)	Yes (*n* = 1306)		No (*n* = 491)	Yes (*n* = 1289)	
*Income activities*									
Extraction income per labourer (PPP$)	149.46	75.13	1.63 ^a^	116.42	78.13	0.89 ^a^	90.51	72.08	0.57 ^a^
	(1404.06)	(512.34)	0.10	(962.59)	(742.18)	0.38	(381.72)	(671.48)	
Days of extraction (number of days)	25.19	24.20	0.41 ^a^	25.85	21.15	1.58 ^a^	21.75	16.81	1.80*^,a^
	(50.14)	(45.46)		(61.04)	(53.19)		(64.03)	(46.42)	
*Demographic characteristics*									
Gender of household head^†^	0.81	0.86	-3.05***^,b^	0.77	0.84	− 3.04***^,b^	0.75	0.82	− 3.08***^,b^
	(0.40)	(0.34)		(0.42)	(0.37)		(0.43)	(0.39)	
Age of household head (years)	54.95	48.28	10.23***^,a^	58.23	51.46	10.11***^,a^	61.21	53.08	12.80***^,a^
	(14.09)	(11.84)		(14.29)	(11.79)		(13.65)	(11.28)	
Household size (persons)	3.66	4.60	− 10.76***^,a^	3.39	4.29	− 9.99***^,a^	2.83	4.17	− 16.62***^,a^
	(1.85)	(1.59)		(1.77)	(1.63)		(1.47)	(1.54)	
Health status of household head^†^	0.66	0.76	− 4.24***^,b^	0.56	0.68	− 4.54***^,b^	0.74	0.86	− 5.94***^,b^
	(0.48)	(0.43)		(0.50)	(0.47)		(0.44)	(0.35)	
Marital status of household head^†^	0.81	0.89	− 4.51***^,b^	0.79	0.86	− 3.25***^,b^	0.77	0.83	− 3.06***^,b^
	(0.39)	(0.31)		(0.41)	(0.35)		(0.42)	(0.38)	
*Human capital*									
Years of schooling of household head (years)	6.54	6.81	− 1.30 ^a^	6.29	6.85	− 2.60***^,a^	6.00	6.94	− 4.52***^,a^
	(3.78)	(4.11)		(3.84)	(4.06)		(3.66)	(4.01)	
Mean schooling years of adult members (years)	5.89	6.07	− 1.20 ^a^	4.69	5.44	− 4.78***^,a^	4.15	5.56	− 9.35***^,a^
	(2.78)	(2.94)		(2.73)	(2.97)		(2.67)	(2.91)	
Share of labourers (%)	73.55	69.64	3.25***^,a^	76.88	73.04	3.09***^,a^	84.82	76.81	6.96***^,a^
	(24.89)	(22.45)		(24.65)	(22.60)		(21.42)	(21.81)	
*Social capital*									
Member of political and social organisation (PSO) ^†^	0.75	0.71	1.82*^,b^	0.77	0.71	2.37**^,b^	0.75	0.69	2.49**^,b^
	(0.43)	(0.45)		(0.42)	(0.45)		(0.44)	(0.47)	
Local households^†^	0.65	0.60	1.68*^,b^	0.62	0.62	− 0.11 ^b^	0.67	0.63	1.33 ^b^
	(0.48)	(0.49)		(0.49)	(0.49)		(0.47)	(0.48)	
*Household assets*									
Land per capita (ha)	0.26	0.24	0.70 ^a^	0.32	0.29	0.79 ^a^	0.39	0.31	2.51**^,a^
	(0.31)	(0.81)		(0.46)	(0.69)		(0.47)	(0.47)	
Asset per capita (PPP$)	589.25	611.97	− 0.49 ^a^	923.87	936.97	− 0.10 ^a^	878.05	1014.75	− 1.60 ^a^
	(702.18)	(955.61)		(3970.67)	(1691.01)		(1088.93)	(1764.66)	
*Physical capital*									
Number of phones	0.78	1.15	− 6.48***^,a^	1.54	2.14	− 7.66***^,a^	1.47	1.88	− 7.21***^,a^
	(0.99)	(1.17)		(1.38)	(1.48)		(1.05)	(1.10)	
Number of tractors	0.39	0.32	2.67***^,a^	0.45	0.38	2.20**^,a^	0.20	0.22	− 0.84 ^a^
	(0.54)	(0.49)		(0.65)	(0.58)		(0.43)	(0.46)	
Number of trucks	0.00	0.01	− 1.51 ^a^	0.00	0.01	− 1.91*^,a^	0.00	0.01	− 2.32**^,a^
	(0.00)	(0.11)		(0.00)	(0.09)		(0.00)	(0.10)	
Number of pushcarts	0.06	0.08	− 1.50 ^a^	0.10	0.13	− 1.25 ^a^	0.33	0.37	− 1.27 ^a^
	(0.24)	(0.29)		(0.30)	(0.38)		(0.59)	(0.63)	
Number of motorcycles	0.74	1.08	− 7.65***^,a^	0.91	1.38	− 8.74***^,a^	1.07	1.69	− 11.16***^,a^
	(0.84)	(0.85)		(0.95)	(1.00)		(1.06)	(1.03)	
*Village characteristics*									
Number of enterprises	0.06	0.13	− 2.74***^,a^	0.85	1.00	− 1.13 ^a^	0.67	0.85	− 1.81**^,a^
	(0.42)	(0.55)		(2.56)	(2.43)		(1.79)	(1.88)	
Share of households with cable internet at home (%)	1.18	1.11	0.39 ^a^	4.30	5.70	− 3.04***^,a^	9.30	11.35	− 2.58**^,a^
	(3.68)	(3.44)		(7.18)	(9.05)		(13.25)	(15.59)	
Having made roads instead of dirt roads^†^	0.18	0.13	2.94***^,b^	0.62	0.66	− 1.63 ^b^	0.91	0.90	0.64 ^b^
	(0.39)	(0.34)		(0.49)	(0.48)		(0.29)	(0.31)	
Distance to province centre (km)	43.97	39.54	3.23***^,a^	43.25	36.66	4.73***^,a^	39.81	37.33	1.83*^,a^
	(26.00)	(26.56)		(26.59)	(25.76)		(25.49)	(25.54)	
Distance to district centre (km)	13.16	12.48	1.38 ^a^	11.93	11.00	2.16**^,a^	11.33	10.87	1.02 ^a^
	(8.82)	(9.69)		(8.30)	(7.95)		(7.96)	(8.92)	
Lagged rainfall (mm)	2521.29	2631.13	− 4.63***^,a^	2346.59	2344.02	0.29 ^a^	2391.33	2369.51	2.52**^,a^
	(432.08)	(466.58)		(162.31)	(168.12)		(163.27)	(162.79)	

Standard deviation in parentheses; ^a^: Two-sample t-test; ^b^: Non-parametric two-sample rank-sum test; †: Dummy; ^∗∗∗^ *p* < 0.01, ^∗∗^ *p* < 0.05, ^∗^ *p* < 0.1

In this paper, besides the instrumental variables from the village data of TVSEP, we also employ the precipitation data from the Tropical Rainfall Measuring Mission (TRMM) which is a joint mission of the National Aeronautics and Space Administration (NASA) and the Japan Aerospace Exploration Agency (JAXA) (see Kummerow et al. (1998) for the details of the TRMM sensors and data algorithms). The precipitation data from TRMM have a high spatial (with 0.25° × 0.25° latitude by longitude) and temporal (recorded daily and 3-hourly) resolution. Since this rainfall data was only available for the period of 1998 to 2014, we use the lagged three-time period (t-3) as an instrumental variable.

Table 1 shows that the income per labourer from resource extraction and the number of days for extraction have been decreasing over time. The number of extraction days decreased from about 24 days in 2010 to about 18 days in 2016. The average income per labourer from extraction activities fell from PPP$ 150 in 2010 to PPP$ 90 in 2016 in the case of households not engaging in non-farm employment. For households engaging in non-farm employment, it decreased slightly from PPP$ 75 in 2010 to PPP$ 72 in 2016. The difference of extraction income between the two groups is not statistically significant.

Households with younger heads and higher schooling years tend to participate more in non-farm employment, while households with a higher share of labourers, and belonging to a political and social organisation (PSO) are not likely to participate in non-farm activities. The difference of household assets between these groups is not statistically significant. Regarding the physical capital, which can be used for non-farm and extraction activities, households engaging in non-farm employment appear to have more phones, trucks, pushcarts, and motorcycles, while households in the other group tend to have more tractors. With regard to village characteristics, the number of enterprises in the villages, the share of households with cable internet at home, and being located closer to the district and province centres are the major differences between the villages of households engaging and not engaging in non-farm activities.

Appendix Table 7 shows the descriptive summary of the household and village characteristics between those participating and not participating in extraction activities in 2010, 2013, and 2016. Regarding the demographic characteristics, in contrast to the descriptive statistics of non-farm participation groups, those households participating in extraction activities are younger, but they have a lower number of schooling years. This indicates that labourers with higher education might have better opportunities to engage in non-farm employment, while lower educated labourers tend to take part in extraction activities.

Households being local (household heads were born in the same as the current village) and with membership in a PSO are less likely to participate in natural extraction activities. The data on household assets point out two important features. First, those households owning more land (related to agricultural production) are more likely to take part in extraction activities. Second, wealthier households (measured by asset value (PPP$) per capita) appear to not likely to get involved in extraction activities.

The descriptive statistics of village characteristics demonstrate the opportunities for non-farm employment such as the number of enterprises in the villages, closer proximity to towns, better infrastructure (e.g. road conditions) and facilities for information and communication technology (ICT) (e.g. cable internet at home) in reducing households’ participation in natural extraction. More importantly, the number of households engaging in extraction activities shows a decreasing trend.

At this point, the question arises whether the available opportunities of non-farm employment play an important role in attracting rural labourers. A higher income from non-farm activities such as self- and wage-employment generates a larger opportunity cost for rural households to participate in resource extraction activities. The annual average income of households not engaging in extraction rose from PPP$ 3,600 in 2010 to PPP$ 4,900 in 2016. These figures are two times higher than that of households engaging in extraction activities in the same period. Hence, solid evidence is needed to validate the role of non-farm employment in helping rural households reduce extraction of natural resources and improve their welfare.

## Econometric specifications

### Identifying the determinants of household participation in rural non-farm employment and non-farm income

As indicated in Fig. 1, various factors of the livelihood platforms influence the choice of a household’s livelihood strategy or activity. A specific activity (e.g. non-farm employment) might not be selected by a number of households as illustrated in Table 1 (the household is a non-participant in non-farm activities). For the other participants who engage in non-farm activities, their non-farm income is positive. This characteristic of the sample enables us to employ the Heckman selection model (Heckman, 1979) to identify factors affecting household’s decision to participate in non-farm employment (the selection stage). The mode is specified as follows:

4 $${S}_{ijt} = {\gamma }_{0}+{\gamma }_{1}{X}_{it}+{\gamma }_{2}{V}_{jt}+{\gamma }_{3}{E}_{jt-3}+{u}_{ijt}$$

In Eq. (4), ${S}_{ijt}$ is the probability for household *i* from village *j* to participate in non-farm employment in year *t*. ${S}_{ijt}$ = 1 if the household participates and = 0 otherwise; ${X}_{it}$ and ${V}_{jt}$ are vectors of household and village characteristics representing the livelihood platform component in Fig. 1; ${\gamma }_{0}$ is the constant, ${\gamma }_{1}$ and ${\gamma }_{2}$ are coefficients of explanatory variables, and ${u}_{ijt}$ is the error term. In addition, as the estimation of the Heckman model should include exclusion restrictions in the selection stage (Sartori, 2003), we use the variable of the lagged three-time period (t-3) precipitation volume as an exclusion restriction as ${\mathrm{E}}_{\mathrm{jt}-3}$ in estimating Eq. (4). We argue that the precipitation reflecting the change in rainfall amount implies the potential of extreme weather events (e.g. drought or heavy rain) which might affect rural households’ livelihoods. Therefore, the participation in non-farm employment in this year could be a result of the previous years’ weather shocks.

In Eq. (4), ${X}_{it}$ (*i* = 1, 2, … 1760) captures demographic characteristics (i.e. household head’s gender, age, household size, health status, and marital status), human capital (i.e. household head’s schooling years, average schooling years of household’s adult members, and share of labourers), and social capital (i.e. being member in a PSO and being local). Besides, we include household financial capital in the model with two indicators, namely land per capita and asset per capita. ${V}_{jt}$ (*j* = 1, 2, … 218) is the group of village characteristics including available non-farm opportunities and local infrastructure such as number of enterprises in the village, distance from village to province centre, share of households with cable internet at home, and villages with made roads (suitable for car/truck access) instead of dirt roads.

For households engaging in non-farm employment, the factors affecting their non-farm income per labourer (the outcome stage) is identified by the equation:

5 $${{\pi }_{\mathrm{non}-\mathrm{farm}}}_{ijt}= {\beta }_{0}+{\beta }_{1}{X}_{it}+{\beta }_{2}{V}_{jt}+ {\varepsilon }_{ijt}$$

In Eq. (5), ${{\uppi }_{\mathrm{non}-\mathrm{farm}}}_{\mathrm{ijt}}$ is the non-farm income per labourer of household *i* from village *j* in year *t*; ${\mathrm{X}}_{\mathrm{it}}$ and ${\mathrm{V}}_{\mathrm{jt}}$ are defined as in Eq. (4); ${\upbeta }_{0}$ is the constant, ${\upbeta }_{1}\mathrm{and}$ ${\upbeta }_{2}$ are associated coefficients of independent variables; and ${\upvarepsilon }_{\mathrm{ijt}}$ is the error term of the household non-farm income equation. Equation 4 and 5 indicate that estimating the Heckman selection model is a two-step procedure. To account for potential biases, the selection parameter (or the inverse Mills ratio) is created from estimating Eq. (4) and is then included in Eq. (5). The inverse Mills ratio (m_i_) for each observation *i* is computed as:

6 $$m_{{\text{i}}} = \frac{{\emptyset \left( {{\text{z}}_{{\text{i}}} \widehat{\gamma }} \right)}}{{\Phi \left( {{\text{z}}_{{\text{i}}} \widehat{\gamma }} \right)}}$$

We run two additional estimations to validate the appropriateness of the exclusion restriction in Eq. (4). The results show that the lagged three-time period precipitation volume only has a significant effect on the participation in non-farm employment, but not on the income from non-farm employment (see Appendix Table 13 for the detailed results). As a robustness check, we run a Probit estimation for Eq. (4) and an Ordinary Least Squares (OLS) regression for Eq. (5). Further, we check for multicollinearity using the Variance Inflation Factor (VIF) values. The VIF values do not show a significant sign of this problem (see Appendix Table 9 for the detailed VIF values). All estimations are bootstrapped with 1000 replications and clustered at commune level to prevent spatial autocorrelation.

### Examining the interrelationship between non-farm income and natural extraction income

Since non-farm employment and natural resource extraction are not dependent but interrelated, they can influence each other. Thus, we use a simultaneous equations model to account for this interdependence. This simultaneous equations model is specified as:

7 $${{\pi }_{\mathrm{non}-\mathrm{farm}}}_{ijt}={\delta }_{0}+{\delta }_{1}{{\pi }_{\mathrm{extract}}}_{it}+{\delta }_{2}{R}_{it}+{\delta }_{3}{X}_{it}+{\delta }_{4}{V}_{jt}+{\mu }_{ijt}$$

8 $${{\pi }_{\mathrm{extract}}}_{ijt} ={\theta }_{0}+{\theta }_{1}{{\pi }_{\mathrm{non}-\mathrm{farm}}}_{it}+{\theta }_{2}{Z}_{it}+{\theta }_{3}{X}_{it}+{\theta }_{4}{V}_{jt}+{\vartheta }_{ijt}$$

In Eq. (7) and (8), ${{\pi }_{\mathrm{non}-\mathrm{farm}}}_{ijt}$ and ${{\pi }_{\mathrm{extract}}}_{ijt}$ refer to incomes per labourer from non-farm and resource extraction activities of household *i* from village *j* in year *t*, respectively. ${X}_{it}$ reflects household’s demographic characteristics, human capital, social capital (as mentioned in Sect. 4.1) and physical capital (including numbers of phones, tractors, trucks, pushcarts, and motorcycles that the household can use for both extraction and non-farm activities). ${V}_{jt}$ denotes village characteristics, namely share of households with cable internet at home and road quality. ${\mu }_{ijt}$ and ${\vartheta }_{ijt}$ are the error terms of non-farm estimation and extraction estimation, respectively.

To address endogeneity between these two key income variables, an instrumental variable (IV) approach is needed for both income variables. We account for this by including ${\mathrm{R}}_{\mathrm{it}}$ and ${\mathrm{Z}}_{\mathrm{it}}$ as IVs for the non-farm income equation (Eq.  7) and the extraction income equation (Eq.  8), respectively. In principle, ${\mathrm{R}}_{\mathrm{it}}$ should have an impact on the non-farm income, but not on the extraction income. Similarly, ${\mathrm{Z}}_{\mathrm{it}}$ should have an impact on the extraction income, but it should not affect the non-farm income. Hence, we use two exogenous variables at village level, namely the distance to province centre and the distance to district centre as IVs in the estimation of non-farm income [as ${\mathrm{R}}_{\mathrm{it}}$ in Eq. (7)] and the number of extraction days as the IV in the estimation of extraction income [as ${\mathrm{Z}}_{\mathrm{it}}$ in Eq. (8)]. For ${\mathrm{R}}_{\mathrm{it}}$ in Eq. (7), the number of extraction days could be used as an IV because some extraction products are only available for fishing, hunting, collecting, or logging in a particular time of the year (e.g. mushroom, fish, and herbs). Therefore, the days of extraction might affect the extraction income, but not the income from non-farm employment. For the ${\mathrm{Z}}_{\mathrm{it}}$ in Eq. (8), the intuition behind these variables is that they represent the opportunity for rural households to engage in non-farm employment. A closer distance to district or province centres means that rural villages provide greater opportunities for off-farm or wage-employment (Nguyen et al., 2021).

Apparently, the inclusion of extraction income per labourer (${{\pi }_{\mathrm{extract}}}_{ijt})$ in the estimation of non-farm income per labourer ${({\pi }_{\mathrm{non}-\mathrm{farm}}}_{ijt})$ and vice versa results in an endogeneity problem. We address this problem by using the three-stage least squares (3SLS) method. This estimation method accounts for the correlation of the endogenous dependent variables and error terms in Eq. (7) and (8). The three steps are well explained by Greene (2018). In the first stage, instrumented values of all endogenous variables are developed (i.e. the predicted values from the regression of each endogenous variable on instrumental variables). In the second stage, consistent estimates for the covariance matrix of the equation disturbances are obtained (these estimates are based on the residuals of the estimations from the estimation of each structure equation in the previous stage). In the last stage, an estimation following the generalised least squares method is performed using the covariance matrix produced in the second stage and instrumented values. Further, the 3SLS method generates more efficient parameter estimates than estimations from two-stage least squares method or a seemingly unrelated regression (Zellner & Theil, 1992).

We run several quality checks of the data and estimation method. First, we check for the problem of multicollinearity by using the VIF values. The results of the VIF values of variables in Eq. (7) and (8) do not show a significant sign of this problem (see Appendix Table 10 for the detailed results of the VIF values). Second, we carry out the Hansen-Sargan overidentification statistical test, the test of independent equations (Breusch-Pagan Lagrange Multiplier Test), the test of overall system heteroscedasticity (Likelihood Ratio LR Test), and the test of overall system heteroscedasticity (Wald Test) to validate the estimation method. The results of these tests confirm the appropriateness of this simultaneous equations model (see Appendix Table 11 for the detailed results of the tests). Third, we run an estimation from a structural equation model as a robustness check. Last, to have robust standard errors and to prevent the spatial autocorrelation, both estimations are bootstrapped with 1000 replications and clustered at commune level.

### Investigating the impacts of non-farm income on household welfare

We examine the impact of non-farm income on both relative and absolute poverty, which is measured in both relative and absolute terms (Foster, 1998). Theoretically, relative poverty is a flexible measurement of poverty using a standard of living in a specific location (e.g. average income of people in the same community) at which people living under that living standard are considered as poor; meanwhile absolute poverty is based on a fixed threshold at which people living under that threshold are considered as poor.

With regard to relative poverty, we calculate the population mean of household income per capita in each year and generate a dummy variable indicating the value of one (1) if the household income per capita falls below 20% of the means; otherwise, the value is zero (0). For absolute poverty, we measure poverty based on Foster et al. (1984) (or the FGT method in short) as:

9 $${P}_{\alpha }=\frac{1}{N}\sum_{i=1}^{H}{\left[\frac{Z- {Y}_{i}}{Z}\right]}^{\alpha }$$

In Eq. (9), P_α_ is the poverty index. The parameter $$\alpha$$ receives the values of 0, 1, and 2 which indicate the poverty headcount ratio (the percentage (incidence) of poverty in a commune), poverty gap (the incidence and the intensity of poverty in a commune), and poverty severity (the incidence, intensity and inequality of the income distribution of the poor households), respectively. Z is the poverty threshold. In this study, we use two different poverty thresholds which are Vietnam’s national poverty level at PPP$ 2.05 per capita a day and the World Bank’s poverty threshold for middle-income countries at PPP$ 3.20 per capita a day (World Bank, 2018). Y_i_ is the daily income per capita of household *i*.

Table 2 shows the description of household income and poverty indicators. With the relative poverty at the 20%-lower-than-the-mean level, there are more households under poverty in both the groups of households participating and not participating in non-farm employment, compared with the figures from the absolute method. Overall, households participating in non-farm employment are less likely to be poor and the poverty indicators of this group show a decreasing trend, while households not participating in non-farm employment have a modest fluctuation in poverty indicators in 2013. We include these poverty indicators as dependent variables in estimations to assess the impacts of non-farm income.

**Tablfe 3:** Poverty indicators of households engaging and not engaging in non-farm employment

	Whole sample (*n* = 5340)	2010		Statistical test	2013		Statistical test	2016		Statistical test
		No (*n* = 525)	Yes (*n* = 1255)		No (*n* = 474)	Yes (*n* = 1306)		No (*n* = 491)	Yes (*n* = 1289)	
Daily per capita income (PPP$)	5.54 (7.40)	3.62 (6.41)	4.24 (6.30)	− 1.91*^,a^	3.78 (7.07)	5.38 (6.30)	− 4.60***^,a^	6.92 (8.41)	7.87 (8.71)	− 2.08***^,a^
Relative poverty^†^	0.59 (0.49)	0.64 (0.48)	0.58 (0.49)	2.14**^,b^	0.75 (0.44)	0.53 (0.50)	8.14***^,b^	0.63 (0.48)	0.56 (0.50)	2.62**^,b^
Absolute poverty at daily per capita income of PPP$ 2.05^†^	0.31 (0.46)	0.46 (0.50)	0.36 (0.48)	4.05***^,b^	0.55 (0.50)	0.28 (0.45)	10.61***^,b^	0.25 (0.43)	0.16 (0.36)	4.53***^,b^
Absolute poverty gap at daily per capita income of PPP$ 2.05	0.18 (0.48)	0.33 (0.68)	0.20 (0.45)	4.88***^,a^	0.39 (0.62)	0.18 (0.55)	7.13***^,a^	0.12 (0.30)	0.07 (0.22)	4.10***^,a^
Absolute poverty severity at daily per capita income of PPP$ 2.05	0.26 (2.98)	0.56 (4.79)	0.24 (2.40)	1.89*^,a^	0.53 (2.47)	0.33 (4.34)	0.96 ^a^	0.11 (0.68)	0.05 (0.42)	1.91**^,a^
Absolute poverty at daily per capita income of PPP$ 3.20^†^	0.47 (0.50)	0.63 (0.48)	0.57 (0.49)	2.36**^,b^	0.69 (0.46)	0.45 (0.50)	9.00***^,b^	0.39 (0.49)	0.28 (0.45)	4.19***^,b^
Absolute poverty gap at daily per capita income of PPP$ 3.20	0.26 (0.42)	0.41 (0.53)	0.30 (0.40)	4.97***^,a^	0.48 (0.50)	0.25 (0.45)	9.42***^,a^	0.19 (0.32)	0.12 (0.25)	4.89***^,a^
Absolute poverty severity at daily per capita income of PPP$ 3.20	0.24 (1.41)	0.45 (2.24)	0.25 (1.16)	2.54**^,a^	0.48 (1.27)	0.26 (2.00)	2.22**^,a^	0.14 (0.43)	0.08 (0.29)	3.35***^,a^

Standard deviation in parentheses; ^a^: Two-sample t-test; ^b^: Non-parametric two-sample rank-sum test; ^†^: Dummy; ^∗∗∗^ *p* < 0.01, ^∗∗^ *p* < 0.05, ^∗^ *p* < 0.1

The model to evaluate the impacts of non-farm income on poverty can be specified as:

10 $${P}_{it}={\varphi }_{0}+{\varphi }_{1}{{\pi }_{\mathrm{non}-\mathrm{farm}}}_{it}+{\varphi }_{2}{X}_{it}+{\omega }_{it}$$

In Eq. (10), $${P}_{it}$$ are poverty indicators including both binary (relative income poor, absolute income poor at PPP$ 2.05, and absolute income poor at PPP$ 3.20) and continuous values (poverty gap at PPP$ 2.05, poverty severity at PPP$ 2.05, poverty gap at PPP$ 3.20, and poverty severity at PPP$ 3.20). $${\varphi }_{0}$$ is the constant. $${X}_{it}$$ includes households’ characteristics that are the same as in the estimations of Eq. (7) and (8) and $${\varphi }_{2}$$ are associated coefficients of households’ variables. $${{\pi }_{\mathrm{non}-\mathrm{farm}}}_{it}$$ is the income per labourer from non-farm employment and $${\varphi }_{1}$$ is its associated coefficient. $${\omega }_{it}$$ is the random error term.

In Eq. (10), the variable $${{\pi }_{\mathrm{non}-\mathrm{farm}}}_{it}$$ is endogenous due to its correlation with other households’ characteristics. We address this problem by using an IV method. More specifically, we use the IV-Probit estimations for binary dependent variables (three dummy variables indicating household income falling below poverty thresholds in both relative and absolute terms) and IV-OLS estimations for continuous dependent variables (four variables capturing poverty gaps and poverty severities at PPP$ 2.05 and PPP$ 3.20 per capita a day).

We employ two exogenous variables at village level that are the distances from the village to district centre and province centre as IVs. Regarding the IV-Probit estimation, we run the Wald tests of exogeneity to validate the appropriateness of the IVs. In the case of the IV-OLS estimation, we run three quality tests, namely the underidentification test, weak identification test, and Sargan-Hansen statistical test for overidentifying restrictions to confirm the appropriateness of these IVs. The results of these IV tests presented on the last four rows of Table 5 validate the appropriateness of these IVs (except for the result of the Wald test of exogeneity in the estimation of absolute income poverty at PPP$ 2.05 per capita a day). As a robustness check for the IV-Probit models, we run three additional estimations with a Recursive Bivariate Probit (RBP) specification for endogenous binary variables (Filippini et al., 2018). In this case, we transform our endogenous variable (non-farm income per labourer) into a dummy indicating the household’s participation in non-farm employment (yes = 1; otherwise = 0). We use the estimation method for a RBP proposed by Coban (2021). Next, we also check for the problem of multicollinearity by using the VIF values. The results of the VIF values do not show a significant sign of this problem (see Appendix Table 12 for the detailed results of the VIF values). Last, all estimations are clustered at the commune level to control for spatial autocorrelation.

## Results and discussion

### Determinants of household’s participation in non-farm employment and non-farm income

Table 3 reports the results of the Heckman selection estimations on the determinants of household’s participation in non-farm employment (selection equation) and income from non-farm activities (outcome equation). The coefficient of Mills ratio is significant and positive implying presence of sample selection bias in the absence of correction (Hoehn, 2006). As a robustness check, the Probit model yields consistent results, while the OLS model shows slightly different results as compared to the Heckman selection model. The determinants with positive impacts include household size, health status of household head, mean schooling years of adult members, share of labourers (in the selection estimation), health status and schooling years of household head, and mean schooling years of adult members (in the outcome estimation). It appears that education of the household head and its members significantly affect household’s decision to participate in non-farm employment as well as its non-farm income. This finding is consistent with Obermann et al. (2020), Lanjouw and Shariff (2004), Rahut and Micevska Scharf (2012), and Rao and Qaim (2011). Therefore, promoting education in rural areas has a positive impact on improving income of local communities. The result that households with more labourers have a higher probability to engage in non-farm activities is in line with Do et al. (2019) and Rahut and Micevska Scharf (2012).

**Table 3:** Household’s decision to participate in non-farm activities and income

	Two-step Heckman selection		Probit		OLS
	Decision to participate	Non-farm income per labourer (ln)	Decision to participate	Marginal effect	Non-farm income per labourer (ln)
Gender of household head^†^	0.093	0.108	0.093		0.449
	(0.095)	(0.116)	(0.095)		(0.409)
Age of household head	− 0.020***	− 0.008	− 0.020***	− 0.006***	− 0.093***
	(0.002)	(0.005)	(0.002)	(0.001)	(0.010)
Household size	0.208***	− 0.004	0.208***	0.060***	0.799***
	(0.025)	(0.045)	(0.025)	(0.007)	(0.084)
Health status of household head^†^	0.119**	0.252***	0.119**	0.034**	0.672***
	(0.052)	(0.080)	(0.052)	(0.015)	(0.223)
Marital status of household head^†^	− 0.182	− 0.051	− 0.182	− 0.053*	− 0.832*
	(0.110)	(0.131)	(0.110)	(0.032)	(0.454)
Schooling years of household head	0.003	0.028***	0.003		0.026
	(0.008)	(0.010)	(0.008)		(0.031)
Mean schooling years of adult members	0.046***	0.048***	0.046***	0.013***	0.211***
	(0.008)	(0.016)	(0.008)	(0.002)	(0.033)
Share of labourers	0.002**	− 0.007***	0.002**	0.001**	0.004
	(0.001)	(0.002)	(0.001)	(0.000)	(0.004)
Member of PSO^†^	− 0.084	− 0.167**	− 0.084		− 0.335
	(0.053)	(0.068)	(0.053)		(0.211)
Local households^†^	− 0.154**	− 0.326***	− 0.154**	− 0.045**	− 0.771**
	(0.073)	(0.098)	(0.073)	(0.021)	(0.315)
Land per capita (ln)	− 0.139***	− 0.195***	− 0.139***	− 0.040***	− 0.611***
	(0.032)	(0.044)	(0.032)	(0.009)	(0.116)
Asset per capita (ln)	− 0.002	0.077	− 0.002		0.059
	(0.030)	(0.093)	(0.030)		(0.168)
Number of enterprises in village	0.039	0.069	0.039		0.189
	(0.050)	(0.051)	(0.050)		(0.186)
Distance to province centre	− 0.004***	− 0.007***	− 0.004***	− 0.001***	− 0.020***
	(0.001)	(0.002)	(0.001)	(0.000)	(0.007)
Share of households with cable internet at home	0.009***	0.015***	0.009***	0.002***	0.038***
	(0.003)	(0.003)	(0.003)	(0.001)	(0.010)
Having made roads instead of dirt roads^†^	0.197***	0.335***	0.197***	0.057***	0.884***
	(0.053)	(0.072)	(0.053)	(0.016)	(0.206)
Lagged rainfall	0.000*		0.000*	0.000*	
	(0.000)		(0.000)	(0.000)	
Constant	− 0.058	6.027***	− 0.058		1.964
	(0.371)	(0.728)	(0.371)		(1.392)
Number of observations	5340		5340		5340
Wald chi2(16)	295.56		385.29		593.94
Prob > chi2	0.000		0.000		0.000
lambda (/mills)	0.878*				
	(0.521)				

Robust standard errors bootstrapped with 1000 replications and clustered at commune level in parentheses; ^†^: Dummy; ln: natural logarithm; ^∗∗∗^ *p* < 0.01, ^∗∗^ *p* < 0.05, ^∗^ *p* < 0.1

In the selection stage, age and marital status of the household head, being local, and land per capita are additional variables that significantly and negatively affect household’s participation. Regarding the outcome stage, variables such as age of household head, share of labourers, being member in a PSO, being local, and land per capita have negative and significant effects on household’s non-farm income per capita. While Do et al. (2019) did not find a significant effect of household head’s age and PSO memberships in the case of Cambodian farmers, our results reveal that these variables significantly influence household’s decision to engage in non-farm activities in Vietnam. The reason is that PSOs play a more important role in Vietnam (especially in rural villages) than in other developing countries because the nation has a strong hierarchical structure of its political and administrative system (Do & Park, 2019). With regard to asset variables, total land area and asset value per capita show neither a significant influence on household’s decision to join non-farm employment nor on non-farm income.

We use several indicators of local infrastructure at village level to examine their impact on rural household’s participation in non-farm activities. This group of village characteristics reveals that the share of households with cable internet at home and better roads have significant and positive impacts on household’s participation and income from non-farm activities. These findings imply that investments in infrastructure and internet development play an important role in providing rural households with opportunities to participate in non-farm employment. Together with an enabling environment, these investments stimulate rural non-farm business development by encouraging self-employment and attracting enterprises to rural regions to increase non-farm incomes and investment (Haggblade et al., 2010).

Besides, our result points out that the distance to the province centre has a significant and negative impact on household’s decision and non-farm income. This finding is in the same vein as that of Babatunde and Qaim (2010), Do et al. (2019), and Senadza (2012) with cases from Nigeria, Cambodia, and Ghana, respectively. This implies that a closer distance from the village to the province centre is significant in providing more opportunities to rural households to engage in non-farm activities and it results in higher incomes.

### Interrelationship between non-farm and natural extraction income

Table 4 stacks the results of the interrelationship between non-farm income and extraction income from the simultaneous equations model and the robustness checks from the structural equations model. It appears that non-farm income and extraction income have a negative and significant relationship. The estimation of the structural equations model also produces a similar result. Furthermore, in the simultaneous estimation model, the income from non-farm activities has a larger magnitude impact on extraction income. In other words, when non-farm employment is available, the opportunity costs of extraction is high. This drives rural households to reduce their participation in extraction activities.

**Table 4:** Determinants of incomes from non-farm activities on natural resource extraction

	Simultaneous estimation model		Structural equations model	
	Extraction income per labourer (ln)	Non-farm income per labourer (ln)	Extraction income per labourer (ln)	Non-farm income per labourer (ln)
Non-farm income per labourer (ln)	− 1.009***		− 0.995**	
	(0.369)		(0.390)	
Extraction income per labourer (ln)		− 0.132***		
		(0.049)		
Days of extraction	0.044***		0.050***	
	(0.006)		(0.003)	
Distance to province centre		− 0.021***		− 0.025***
		(0.006)		(0.006)
Distance to district centre		− 0.010		− 0.012
		(0.014)		(0.015)
Gender of household head^†^	0.929*	0.439	0.900*	0.342
	(0.502)	(0.388)	(0.529)	(0.394)
Age of household head	− 0.137***	− 0.103***	− 0.133***	− 0.096***
	(0.039)	(0.010)	(0.040)	(0.010)
Household size	0.884***	0.729***	0.856***	0.687***
	(0.278)	(0.078)	(0.284)	(0.079)
Health status of household head^†^	0.227	0.295	0.218	0.302
	(0.300)	(0.227)	(0.316)	(0.236)
Marital status of household head^†^	− 1.302**	− 1.059**	− 1.294*	− 1.029**
	(0.653)	(0.452)	(0.684)	(0.455)
Schooling years of household head	− 0.190***	− 0.039	− 0.181***	− 0.007
	(0.038)	(0.029)	(0.040)	(0.029)
Mean schooling years of adult members	0.191**	0.170***	0.196**	0.176***
	(0.082)	(0.035)	(0.088)	(0.035)
Share of labourers	− 0.013*	− 0.009*	− 0.013*	− 0.009*
	(0.007)	(0.005)	(0.007)	(0.005)
Member of PSO^†^	− 0.284	− 0.267	− 0.301	− 0.286
	(0.258)	(0.211)	(0.269)	(0.213)
Local households^†^	− 0.588	− 0.240	− 0.599	− 0.204
	(0.390)	(0.300)	(0.404)	(0.301)
Number of phones	0.345*	0.368***	0.343*	0.376***
	(0.183)	(0.080)	(0.196)	(0.082)
Number of tractors	− 1.268**	− 1.376***	− 1.219**	− 1.365***
	(0.582)	(0.211)	(0.605)	(0.217)
Number of trucks	− 0.043	1.925***	0.028	2.329***
	(1.035)	(0.515)	(1.122)	(0.560)
Number of pushcarts	0.123	0.033	0.132	0.028
	(0.257)	(0.196)	(0.268)	(0.194)
Number of motorcycles	0.397	0.672***	0.405	0.732***
	(0.304)	(0.123)	(0.328)	(0.122)
Share of households with cable internet at home	0.001	0.026***	0.002	0.031***
	(0.019)	(0.009)	(0.019)	(0.009)
Having made roads instead of dirt roads^†^	− 0.641*	0.307	− 0.639	0.459**
	(0.386)	(0.229)	(0.394)	(0.209)
Constant	4.779**	5.337***	4.454**	5.192***
	(1.872)	(1.000)	(1.907)	(1.004)
Number of observations	5340		5340	
Wald chi2	400.47		512.77	
Prob > chi2	0.000		0.000	

Robust standard errors bootstrapped with 1000 replications and clustered at commune level in parentheses; ^†^: Dummy; ln: natural logarithm; ^∗∗∗^ *p* < 0.01, ^∗∗^ *p* < 0.05, ^∗^ *p* < 0.1

**Table 5:** Impacts of non-farm income on poverty

	Relative poverty	Absolute poverty at PPP$ 2.05 per day			Absolute poverty at PPP$ 3.20 per day		
	Income poor	Income poor	Poverty gap	Poverty severity	Income poor	Poverty gap	Poverty severity
Non-farm income per labourer (ln)	− 0.110***	− 0.143***	− 0.046***	− 0.037	− 0.099***	− 0.044***	− 0.042**
	(0.037)	(0.025)	(0.013)	(0.033)	(0.038)	(0.013)	(0.019)
Gender of household head^†^	− 0.117	− 0.165*	− 0.016	0.047	− 0.156	− 0.026	0.006
	(0.092)	(0.097)	(0.025)	(0.050)	(0.100)	(0.025)	(0.031)
Age of household head	− 0.010**	− 0.018***	− 0.005***	− 0.004	− 0.013***	− 0.006***	− 0.005**
	(0.004)	(0.003)	(0.001)	(0.003)	(0.004)	(0.001)	(0.002)
Household size	0.257***	0.228***	0.053***	0.026	0.264***	0.063***	0.046*
	(0.021)	(0.019)	(0.012)	(0.056)	(0.022)	(0.011)	(0.027)
Health status of household head^†^	− 0.086	− 0.129**	− 0.035**	0.007	− 0.177***	− 0.046***	− 0.021
	(0.058)	(0.052)	(0.016)	(0.077)	(0.061)	(0.014)	(0.037)
Marital status of household head^†^	− 0.011	0.026	0.001	0.034	0.092	0.006	0.016
	(0.120)	(0.120)	(0.034)	(0.058)	(0.134)	(0.035)	(0.041)
Years of schooling of household head	− 0.022***	− 0.015*	− 0.001	0.011	− 0.017**	− 0.003	0.003
	(0.008)	(0.008)	(0.002)	(0.010)	(0.008)	(0.002)	(0.005)
Mean schooling years of adult members	− 0.025**	− 0.001	− 0.002	− 0.017	− 0.013	− 0.002	− 0.008
	(0.012)	(0.010)	(0.003)	(0.018)	(0.012)	(0.003)	(0.009)
Share of labourers	− 0.005***	− 0.004***	− 0.001*	− 0.000	− 0.004***	− 0.001***	− 0.001
	(0.001)	(0.001)	(0.000)	(0.003)	(0.001)	(0.000)	(0.001)
Member of PSO^†^	− 0.007	− 0.039	− 0.001	− 0.045	0.022	− 0.002	− 0.020
	(0.046)	(0.042)	(0.016)	(0.125)	(0.052)	(0.014)	(0.057)
Local households^†^	0.192***	0.093	− 0.021	− 0.079	0.082	− 0.004	− 0.038
	(0.064)	(0.060)	(0.020)	(0.103)	(0.063)	(0.018)	(0.050)
Number of phones	− 0.010	− 0.020	0.007	0.047	− 0.070**	− 0.002	0.020
	(0.026)	(0.025)	(0.008)	(0.038)	(0.030)	(0.007)	(0.019)
Number of tractors	− 0.294***	− 0.197***	− 0.053**	0.020	− 0.158**	− 0.058**	− 0.025
	(0.057)	(0.064)	(0.025)	(0.112)	(0.072)	(0.025)	(0.054)
Number of trucks	− 0.280	− 0.564	0.002	− 0.187	− 0.357	− 0.028	− 0.084
	(0.353)	(0.456)	(0.039)	(0.132)	(0.403)	(0.043)	(0.068)
Number of pushcarts	− 0.015	− 0.184***	− 0.035**	− 0.128*	− 0.257***	− 0.046***	− 0.077**
	(0.040)	(0.062)	(0.016)	(0.072)	(0.055)	(0.014)	(0.036)
Number of motorcycles	− 0.238***	− 0.216***	− 0.000	0.067	− 0.321***	− 0.028**	0.018
	(0.078)	(0.075)	(0.014)	(0.055)	(0.076)	(0.014)	(0.028)
Constant	1.180***	0.898***	0.534***	0.355	1.048***	0.662***	0.505**
	(0.229)	(0.222)	(0.102)	(0.486)	(0.241)	(0.099)	(0.240)
Number of observations	5340	5340	5340	5340	5340	5340	5340
Wald chi2(16)	1067.75	1656.99	253.69	83.33	913.67	511.63	199.98
Prob > chi2	0.000	0.000	0.000	0.000	0.000	0.000	0.000
Wald test of exogeneity (Prob > chi2)	0.105	0.010			0.194		
Underidentification			0.000	0.000		0.000	0.000
Weak identification			40.536	40.536		40.536	40.536
Overidentification			0.545	0.147		0.567	0.167

Robust standard errors clustered at commune level in parentheses; ^†^: Dummy; ln: natural logarithm; ^∗∗∗^ *p* < 0.01, ^∗∗^ *p* < 0.05, ^∗^ *p* < 0.1. The underidentifying test is an LM test relied on the rk LM statistics (Kleibergen & Paap, 2006) with the null hypothesis stating that the model is underidentified. The overidentifying test based on the Hansen J test with the null hypothesis stating that all instruments are valid in the model. The reported values of underidentification and overidentification tests are p-values. The report of weak-identifying test uses the Kleibergen-Paap rk Wald F statistics

Regarding the estimation of extraction income, the results indicate that gender of the household head, household size, mean schooling years of adult members in the household, and number of phones have a positive and significant effect on income from the extraction of natural resources. On the contrary, household head’s age, marital status and number of schooling years, the share of labourers, and the number of tractors have negative and significant effects on the extraction income. These findings are in line with Nguyen, Do, et al. (2015) who found that younger household heads are more likely to rely on extraction income due to their lower level of experience and the education level of households has a negative effect on environmental resource extraction. Further, improving the educational level of the household head is important to reduce extraction of natural resources. We, therefore, recommend that the promotion of education and local government’s policies should also consider environmental awareness of villages in rural areas. Our recommendation is in the same direction as proposed by Gkargkavouzi et al. (2019) who suggest putting more emphasis on knowledge, constraints and motives to environmental behaviour in designing policy interventions.

With regard to the non-farm income estimation, there are some similarities to the results of the extraction income in terms of impact direction and significance. Variables with negative impacts include age and marital status of household heads, share of labourers, and number of tractors, and variables with positive impacts consist of household size, mean schooling years of adult members, and number of phones. One difference in the group of physical capital variables is the positive and significant effect of number of trucks and motorcycles. Evidently, these vehicles might have a positive relationship with non-farm activities rather than extraction activities. This finding stands in contrast to that of Nguyen et al. (2018b) with the case of Cambodian households. The reason is that rural households in Cambodia strongly rely on extraction income (Nguyen, Do, et al., 2015). Overall, the results remain consistent with the estimation on the determinants of household’s participation in non-farm employment and non-farm income in the previous section.

In addition, our result also shows a significant and positive impact of cable internet at home on non-farm income and a significant and negative impact of better road conditions on natural resource extraction income. The effect of road quality on reducing extraction income is in the same vein as that of Nguyen, Do, et al. (2015) with the case of rural households in Cambodia. Although the impact of the better road variable on non-farm income is not statistically significant, its negative impact on extraction income in our simultaneous estimation implies that non-farm employment can be an alternative for income diversification of rural households. Thus, improvements in infrastructure and facilities of ICT play an important role in discouraging household’s participation in natural resource extraction and stimulating rural villagers to engage in non-farm work.

### Impacts of non-farm income on poverty

Table 5 presents the impacts of non-farm income on poverty indices. It appears that income from non-farm activities can significantly reduce poverty in both relative and absolute terms and in several different aspects such as head count ratio, poverty gap, and poverty severity. As a robustness check, the results from the RBP models show that household’s participation in non-farm employment has a negative correlation with poverty in relative term and absolute term at daily income of PPP$ 2.05 per capita (see Appendix Table 14 for the detailed results). Some scholars have found that non-farm income could help improve food security (Do et al., 2019) and reduce food poverty (Bui & Hoang, 2021), our findings complement the role of non-farm employment by adding a comprehensive view on improving rural household welfare and poverty reduction in particular. This relationship between non-farm income and poverty reduction points to the need of an appropriate environment for providing non-farm opportunities for rural households. This implication is in the same vein as the recommendation of Haggblade et al. (2010). Furthermore, in the current context of extraordinary event such as the Covid-19 Pandemic, the stimulation of improving rural economies is more vital when migration and remittances are affected significantly (Waibel et al., 2020).

Our results signify that rural-based manufacturing or self-businesses with better income opportunities can significantly contribute to rural poverty alleviation. However, the constraints of poor infrastructure in rural areas needs to be addressed (Fan et al., 2002). In this regard, government spending on improving education (e.g. technical training) and infrastructure (e.g. better roads) can positively affect rural household income (Do & Park, 2019; Hoang, 2020). Hence, investments in rural regions need to be stimulated to provide rural non-farm opportunities.

## Summary and conclusion

Given the problem of over-exploitation and alarming depletion of natural resources in developing countries, we identify the determinants of rural households’ participation in non-farm employment as an alternative livelihood strategy for rural households, especially for the poor who are significantly relying on income from natural resource extraction. We examine the interrelationship between non-farm employment and natural resource extraction. Lastly, we investigate the impacts of non-farm income on rural households’ welfare and poverty reduction in particular. In this study, we use a dataset of 1780 identical households from Vietnam collected in 2010, 2013, and 2016 to address the above research questions.

To address the first research question, we use the two-step Heckman selection model to prevent the potential problem of selection biases. Our findings reveal that household’s characteristics including household size, mean schooling years of adult members, health status, schooling years of household head, and share of household’s labourers have positive and significant effects, while age, marital status of household head, household’s membership in PSO, and local households have significant and negative influences on household’s participation in non-farm employment and non-farm income. Among village characteristics, we find that cable internet at home and a higher quality of roads positively affect households’ participation and income from non-farm activities.

With regard to the second research problem, the results from the simultaneous estimation model show that non-farm income and income from natural resource extraction have a negative inter-correlation. Besides, a larger magnitude impact of income from non-farm activities on extraction income implies that, when the opportunities of non-farm employment is available, they might drive rural households to reduce their participation in extraction activities. We also find that local households have a lower level of natural extraction incomes. Our results further reveal that there is a significant and positive impact of cable internet at home on non-farm income and a significant and negative impact of better road conditions on extraction income.

We use both relative and absolute poverty to examine the impact of non-farm income on poverty reduction. Our findings show that non-farm activities significantly reduce poverty in both relative and absolute terms. This is true for the headcount ratio, the poverty gap, and poverty severity. Further, the results in this section show that there is a need of an appropriate environment to provide non-farm opportunities for rural households.

These findings from our study lead to several important policy implications. First, the relationship of the educational level of the household head with extraction income is significantly negative. Therefore, promoting education in rural areas is important and can have positive impacts on the sustainability of local communities. Second, provincial authorities should put more emphasis on supporting local households and small landholders who are less likely to engage in non-farm activities. Last, investments in infrastructure development and ICT facilities play an important role in providing rural households with opportunities to engage in non-farm employment. Together with an enabling environment, these investments stimulate rural non-farm business development by encouraging self-employment and attracting enterprises to rural regions to increase non-farm incomes and investment. Hence, the stimulation of rural development policies on providing non-farm opportunities is critical to help to increase income, improve welfare, and, more importantly, reduce natural resource exploitation of rural households in developing countries such as Vietnam.

Although our study provides some important insights, it still has a few limitations. We could not take advantage of panel data since we used estimation methods for a pooled sample. These methods could not allow us to control for the unobservable characteristics of each household. Besides, the use of pooled data might cause the problem of heteroscedasticity.

## Figures and Tables

**Table 7:**
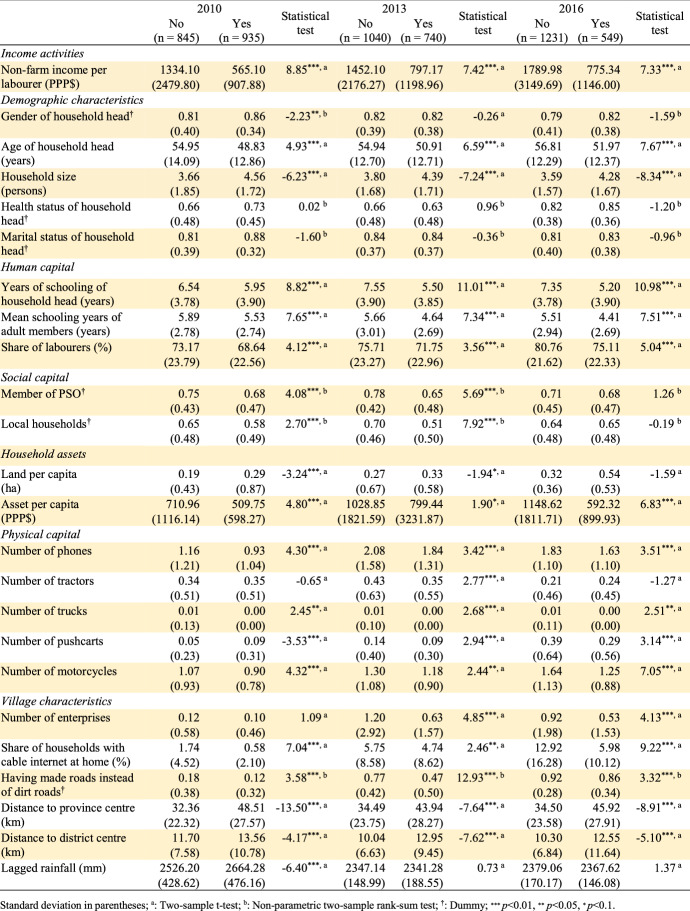
Descriptive summary of household characteristics by participation in extraction activities

**Table 8:**
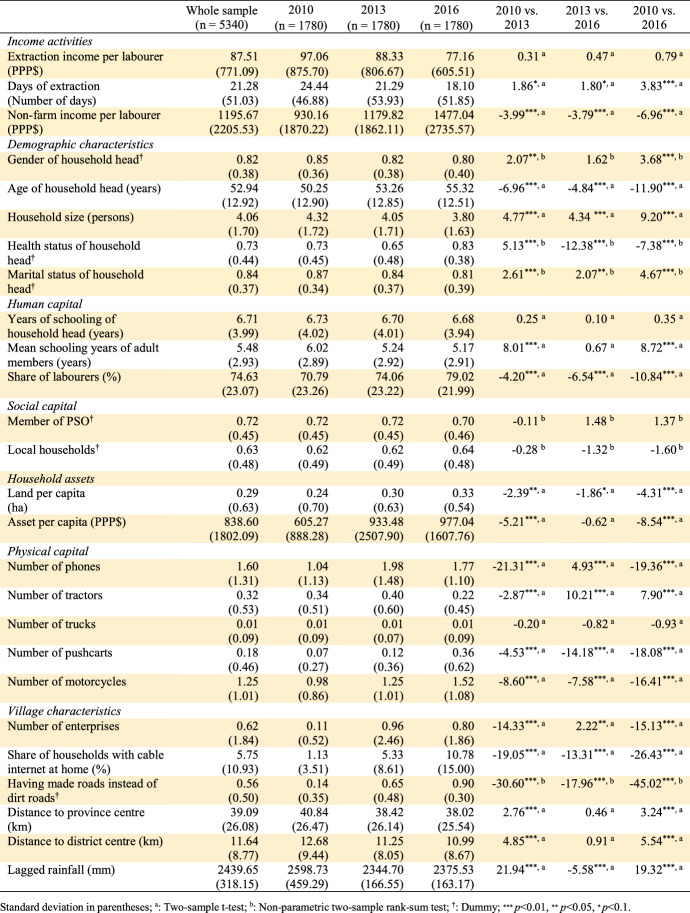
Descriptive summary of household’s characteristics by years

## Appendices

See Tables 6, 7, 8, 9, 10, 11, 12, 13, 14.

**Table 6:** Definition and measurement of household and village characteristics

Variables	Measurement	Definition
*A. Income activities*		
Extraction income per labourer	PPP$ (adjusted to 2005 prices)	Household income per labour member from natural resource extraction
Days of extraction	(Number of days)	Number of days that the household can extract each year
Non-farm income per labourer	PPP$ (adjusted to 2005 prices)	Household income per labour member from non-farm wage- and self-employment activities
*B. Demographic characteristics*		
Gender of household head	Dummy	Gender of the household head. Male household head = 1; otherwise = 0
Age of household head	Years	Age of the household head
Household size	Number of persons	Number of members in the household
Health status of household head	Dummy	If household head is healthy or “can manage” = 1; otherwise = 0
Marital status of household head	Dummy	If the household head is married. Yes = 1; otherwise = 0
*C. Human capital*		
Years of schooling of household head	Years	Number of schooling years of the household head
Mean schooling years of adult members	Years	Average years of schooling of adult members in the household
Share of labourers	Percentage (%)	Share of members in working ages (from 15 to 64 years old) in the household
*D. Social capital*		
Member of political and social organisation (PSO)	Dummy	If the household head is a member of political or social organisation = 1; otherwise = 0
Local household	Dummy	If the household head was born in the same as the current village = 1; otherwise = 0
*E. Household assets*		
Household land area per capita	hectares (ha)	Land area per capita of the household
Household asset value per capita	PPP$ (adjusted to 2005 prices)	Total asset values per capita of the household include productive and non-productive assets
*F. Physical capital*		
Number of phones	Quantity	Number of phones that the household owns
Number of tractors	Quantity	Number of 2-wheels and 4-wheels tractors that the household owns
Number of trucks	Quantity	Number of trucks that the household owns
Number of pushcarts	Quantity	Number of pushcarts that the household owns
Number of motorcycles	Quantity	Number of motorcycles that the household owns
*G. Village characteristics*		
Number of enterprises	Quantity	Number of enterprises with more than nine employees in the village
Share of households with cable internet at home	Percentage (%)	The percentage of households with cable internet at home in the village
Having made roads instead of dirt roads	Dummy	If the main roads in the village are made roads (instead of dirt roads) = 1; otherwise = 0
Distance to province centre	Kilometre (km)	The distance from the village to the province centre
Distance to district centre	Kilometre (km)	The distance from the village to the district centre
Lagged rainfall	Millimetre (mm)	The total annual amount of precipitation in lagged three-time period at the village level

**Table 9:** Variance inflation factor values on the estimations of participation in non-farm employment and non-farm income

Variable	Participation in non-farm employment	Income from non-farm employment
Gender of household head^†^	3.06	3.06
Age of household head	1.31	1.31
Household size	1.36	1.36
Health status of household head^†^	1.21	1.20
Marital status of household head^†^	3.17	3.17
Years of schooling of household head	1.83	1.82
Mean schooling years of adult members	1.68	1.68
Share of labourers	1.25	1.25
Member of PSO^†^	1.12	1.10
Local household^†^	1.22	1.20
Land per capita (ln)	1.18	1.17
Household asset value per capita (ln)	1.35	1.34
Number of enterprises	1.06	1.06
Share of households with cable internet at home	1.16	1.16
Having made roads instead of dirt roads^†^	1.23	1.18
Distance to province centre	1.16	1.16
Lagged rainfall	1.11	
Mean VIF	1.50	1.51

^†^: Dummy; ln: natural logarithm

**Table 10:** Variance inflation factor values on the simultaneous estimation model

Variables	Non-farm income	Extraction income
Extraction income per labourer (ln)	1.22	
Distance to province centre	1.21	
Distance to district centre	1.13	
Non-farm income per labourer (ln)		1.21
Days of extraction		1.07
Gender of household head^†^	3.06	3.05
Age of household head	1.33	1.36
Household size	1.51	1.55
Health status of household head^†^	1.17	1.17
Marital status of household head^†^	3.18	3.18
Years of schooling of household head	1.86	1.83
Mean schooling years of adult members	1.68	1.68
Share of labourers	1.28	1.28
Member of PSO^†^	1.09	1.09
Local household^†^	1.19	1.13
Number of phones	1.48	1.48
Number of tractors	1.06	1.08
Number of trucks	1.01	1.01
Number of pushcarts	1.08	1.08
Number of motorcycles	1.65	1.65
Share of households with cable internet at home	1.17	1.15
Having made roads instead of dirt roads^†^	1.27	1.25
Mean VIF	1.48	1.49

^†^: Dummy; ln: natural logarithm

**Table 11:** Quality tests of the simultaneous estimation model

	chi2	Prob. > chi2
Hansen-Sargan overidentification statistics	1.933	0.164
Tests of independent equations (Breusch-Pagan Lagrange Multiplier Test)	3908.197	0.000
Tests of Overall System Heteroscedasticity (Likelihood Ratio LR Test)	7028.994	0.000
Tests of Overall System Heteroscedasticity (Wald Test)	4572.613	0.000

**Table 12:** Variance Inflation Factor values on the estimations of non-farm income and poverty

Variables	VIF	1/VIF
Non-farm income per labourer (ln)	1.23	0.81
Gender of household head^†^	3.05	0.33
Age of household head	1.34	0.75
Household size	1.54	0.65
Health status of household head^†^	1.17	0.85
Marital status of household head^†^	3.18	0.31
Years of schooling of household head	1.81	0.55
Mean schooling years of adult members	1.67	0.60
Share of labourers	1.28	0.78
Member of PSO^†^	1.09	0.92
Local household^†^	1.10	0.91
Number of phones	1.45	0.69
Number of tractors	1.07	0.94
Number of trucks	1.01	0.99
Number of pushcarts	1.05	0.95
Number of motorcycles	1.63	0.61
Distance to province centre	1.17	0.85
Distance to district centre	1.12	0.90
Mean VIF	1.50	

^†^: Dummy; ln: natural logarithm

**Table 13:** Estimations on validating the exclusion restriction for the Heckman selection model

	Probit	OLS
	Decision to participate	Non-farm income per labourer (ln)
Gender of household head^†^	0.093	0.441
	(0.095)	(0.408)
Age of household head	− 0.020***	− 0.093***
	(0.002)	(0.010)
Household size	0.208***	0.797***
	(0.025)	(0.083)
Health status of household head^†^	0.119**	0.638***
	(0.052)	(0.228)
Marital status of household head^†^	− 0.182	− 0.817*
	(0.110)	(0.454)
Schooling years of household head	0.003	0.027
	(0.008)	(0.031)
Mean schooling years of adult members	0.046***	0.213***
	(0.008)	(0.033)
Share of labourers	0.002**	0.004
	(0.001)	(0.004)
Member of PSO^†^	− 0.084	− 0.377*
	(0.053)	(0.213)
Local households^†^	− 0.154**	− 0.814***
	(0.073)	(0.314)
Land per capita (ln)	− 0.139***	− 0.605***
	(0.032)	(0.116)
Asset per capita (ln)	− 0.002	0.065
	(0.030)	(0.168)
Number of enterprises in village	0.039	0.188
	(0.050)	(0.190)
Distance to province centre	− 0.004***	− 0.020***
	(0.001)	(0.007)
Share of households with cable internet at home	0.009***	0.039***
	(0.003)	(0.010)
Having made roads instead of dirt roads^†^	0.197***	0.955***
	(0.053)	(0.220)
*Exclusion restriction variable*		
Lagged rainfall	0.000*	0.001
	(0.000)	(0.000)
Constant	− 0.058	0.693
	(0.371)	(1.661)
Number of observations	5340	5340
Wald chi2(17)	385.29	595.62
Prob > chi2	0.000	0.000

Robust standard errors bootstrapped with 1000 replications and clustered at commune level in parentheses; ^†^: Dummy; ln: natural logarithm; ^∗∗∗^ *p* < 0.01, ^∗∗^ *p* < 0.05, ^∗^ *p* < 0.1

**Table 14:** Results from Recursive Bivariate Probit estimations on household’s participation in non-farm employment and poverty (outcome equation)

	Relative poverty	Income poverty at PPP$ 2.05 per capita a day	Income poverty at PPP$ 3.20 per capita a day
Participation in non-farm employment^†^	− 0.970*	− 0.855**	− 0.492
	(0.579)	(0.426)	(0.451)
Gender of household head^†^	− 0.126	− 0.204**	− 0.168**
	(0.086)	(0.103)	(0.083)
Age of household head	− 0.007*	− 0.012***	− 0.008**
	(0.004)	(0.004)	(0.004)
Household size	0.247***	0.206***	0.233***
	(0.033)	(0.026)	(0.029)
Health status of household head^†^	− 0.103	− 0.184***	− 0.201***
	(0.065)	(0.052)	(0.060)
Marital status of household head^†^	0.033	0.132	0.163
	(0.105)	(0.118)	(0.115)
Schooling years of household head	− 0.025***	− 0.020**	− 0.019**
	(0.008)	(0.009)	(0.008)
Mean schooling years of adult members	− 0.034***	− 0.019*	− 0.025**
	(0.009)	(0.010)	(0.010)
Share of labourers	− 0.004***	− 0.003***	− 0.004***
	(0.001)	(0.001)	(0.001)
Member of PSO^†^	0.009	− 0.006	0.046
	(0.058)	(0.065)	(0.063)
Local households^†^	0.192***	0.095*	0.074
	(0.055)	(0.056)	(0.053)
Number of phones	− 0.029	− 0.068**	− 0.101***
	(0.026)	(0.028)	(0.026)
Number of tractors	− 0.225***	− 0.068	− 0.059
	(0.071)	(0.050)	(0.059)
Number of trucks	− 0.494	− 1.035	− 0.583
	(0.387)	(1.868)	(0.818)
Number of pushcarts	− 0.028	− 0.245***	− 0.282***
	(0.039)	(0.058)	(0.059)
Number of motorcycles	− 0.306***	− 0.364***	− 0.402***
	(0.045)	(0.035)	(0.035)
Constant	1.529***	1.074***	1.072***
	(0.483)	(0.397)	(0.414)
Number of observations	5340	5340	5340
Wald chi2(33)	4893.00	5908.05	3456.39
Prob > chi2	0.000	0.000	0.000

Robust standard errors with 50 replications and clustered at commune level in parentheses; ^†^: Dummy; ln: natural logarithm; ^∗∗∗^ *p* < 0.01, ^∗∗^ *p* < 0.05, ^∗^ *p* < 0.1
